# Cyclohexyl-griselimycin Is Active against Mycobacterium abscessus in Mice

**DOI:** 10.1128/AAC.01400-21

**Published:** 2022-01-18

**Authors:** Wassihun Wedajo Aragaw, Christine Roubert, Evelyne Fontaine, Sophie Lagrange, Matthew D. Zimmerman, Véronique Dartois, Martin Gengenbacher, Thomas Dick

**Affiliations:** a Center for Discovery and Innovation, Hackensack Meridian Health, Nutley, New Jersey, USA; b Evotec ID (Lyon) SAS, Lyon, France; c Department of Medical Sciences, Hackensack Meridian School of Medicine, Nutley, New Jersey, USA; d Department of Microbiology and Immunology, Georgetown University, Washington, DC, USA

**Keywords:** *Mycobacterium abscessus*, nontuberculous mycobacteria, NTM, griselimycin, DnaN

## Abstract

Cyclohexyl-griselimycin is a preclinical candidate for use against tuberculosis (TB). Here, we show that this oral cyclodepsipeptide is also active against the intrinsically drug-resistant nontuberculous mycobacterium Mycobacterium abscessus *in vitro* and in a mouse model of infection. This adds a novel advanced lead compound to the M. abscessus drug pipeline and supports a strategy of screening chemical matter generated in TB drug discovery efforts to fast-track the discovery of novel antibiotics against M. abscessus.

## INTRODUCTION

Mycobacterium abscessus causes difficult-to-cure lung disease. Multidrug regimens are administered for months to years and typically contain an oral macrolide (azithromycin or clarithromycin) and intravenously administered amikacin, imipenem/cefoxitin, or tigecycline. However, cure rates are low (<50%), and patients often undergo surgical lung resection, if feasible (1–3). Given the poor performance of the current regimens, more efficacious drugs are needed. Not surprisingly, the M. abscessus drug pipeline is thinly populated and largely focused on repurposing and reformulation of approved antibiotics. *De novo* drug discovery efforts (new chemotypes and/or new targets) are hindered by extremely low hit rates in screens attempting to identify chemical starting points (4, 5).

M. abscessus is intrinsically resistant to many antituberculosis (anti-TB) antibiotics, including all first-line drugs (6). Despite M. abscessus resistance to most approved antituberculars, we found that compound collections of TB actives provide a rich source for the identification of hits against M. abscessus (7). In contrast to the limited efforts in M. abscessus drug discovery, anti-TB drug discovery experienced a renaissance over the past 2 decades, resulting in a number of advanced lead series (Stop TB Working Group on New TB Drugs [https://www.newtbdrugs.org/pipeline/discovery]). The mechanism of action of many anti-TB leads has been elucidated, and pharmacokinetic (PK) properties have been optimized to enable proof-of-concept studies in animal models. Prioritization of advanced TB leads avoids the high attrition encountered in early lead optimization due to failure to introduce favorable PK properties and thus should accelerate the drug discovery process for M. abscessus. To leverage these advances, we screened TB leads against M. abscessus and identified several novel anti-M. abscessus compounds with demonstrated *in vivo* activity, including inhibitors of ATP synthase (8), leucyl tRNA synthetase (9, 10), and DNA gyrase (11). Expanding on this strategy, we asked whether the recently identified preclinical anti-TB candidate cyclohexyl-griselimycin (CGM) (12) is active against M. abscessus.

**FIG 1:**
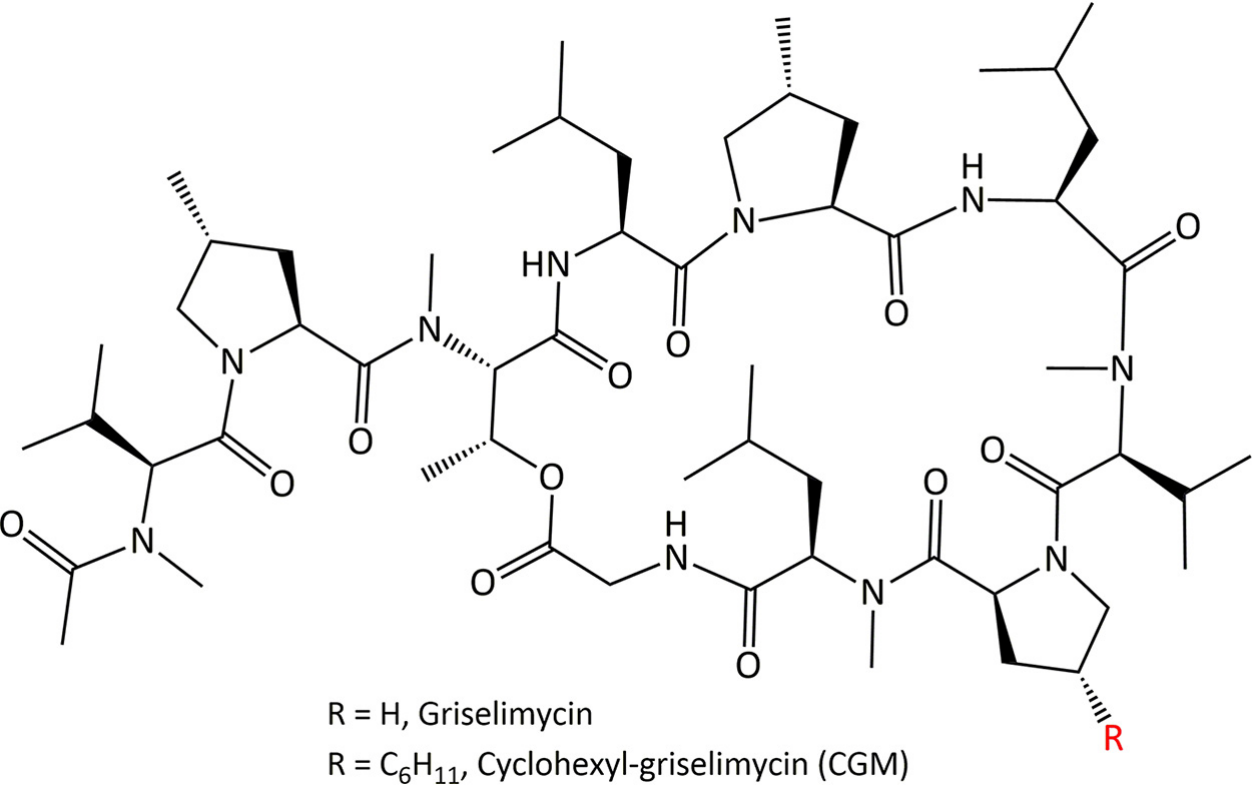
Structure of cyclohexyl-griselimycin, CGM (12).

Griselimycins are cyclic depsipeptides that were originally isolated from *Streptomyces* species (13). Evidence for the anti-TB activity of these natural products goes back to their discovery in the 1960s. However, the first human studies were halted due to poor oral bioavailability (12, 14, 15). These forgotten natural products were recently revisited by investigators from Sanofi in association with TB Alliance to identify analogs with improved PK properties. The cyclohexyl analog CGM (Fig. 1) showed excellent *in vitro* potency and attractive oral bioavailability and efficacy in TB mouse models (12). Interestingly, resistance against this new drug candidate emerged at extremely low frequency and was associated with strong fitness costs (12). Genome analyses revealed that resistance was associated with amplification of large chromosomal segments, all containing the *dnaN* gene, suggesting *dnaN* overexpression as a mechanism of resistance (12). Indeed, binding studies and costructural analyses showed that griselimycins target mycobacterial DnaN (12).

DnaN encodes the DNA sliding clamp, also referred to as DNA polymerase III β subunit. This DNA sliding clamp is crucial for bacterial DNA replication and repair, acting as a protein-protein interaction (PPI) hub. The protein surrounds double-stranded DNA and functions to recruit a diverse range of accessory proteins involved in DNA metabolism (16–18). DnaN protein partners interact with a specific hydrophobic cleft on the DnaN clamp. Griselimycins bind to the same cleft, disrupting DnaN PPIs, as shown by elegant biochemical analyses (12). On-target activity was recently confirmed by de Wet and colleagues in intact mycobacteria (19). By combining inducible CRISPR interference and image-based analyses of morphological features in mycobacteria, the authors demonstrated that griselimycin copied the phenotype of a *dnaN* knockdown (19). Fluorescence microscopy analyses further demonstrated that griselimycins cause replisome instability and affect the structure of the nucleoid *in vivo* (20). Thus, the peptide antibiotic griselimycin corrupts DnaN-dependent machines involved in genome copying and maintenance by acting as a PPI inhibitor.

Interestingly, CGM not only was potent *in vitro* against Mycobacterium tuberculosis but also was active against the nonpathogenic mycobacterial model organism Mycobacterium smegmatis (12). To determine whether CGM retained activity against the opportunistic pathogen M. abscessus, we measured its MIC against reference strains and clinical isolates of the three M. abscessus complex subspecies, using CGM from Evotec’s compound archive (12). Dose-response curves were established using the broth dilution method in Middlebrook 7H9 medium (BD Difco) and optical density at 600 nm (OD_600_) as the readout for growth (21). CGM exhibited uniform submicromolar growth-inhibitory activity against all M. abscessus strains tested (Table 1), suggesting that CGM is broadly active against the M. abscessus complex.

**TABLE 1:** *In vitro* activity of cyclohexyl-griselimycin (CGM) against M. abscessus

Strains^*b*^	*erm*(41) sequevar^*c*^	CLR sensitivity^*c*^	MIC (μM)^*a*^	
			CGM	CLR
Culture collection reference strains				
M. abscessus subsp. *abscessus* ATCC 19977	T28	Resistant	0.5	3
M. abscessus subsp. *massiliense* CCUG 48898T	Deletion	Sensitive	0.8	0.8
M. abscessus subsp. *bolletii* CCUG 50184T	T28	Resistant	0.8	6

Clinical isolates^*b*^				
M. abscessus subsp. *abscessus* bamboo	C28	Sensitive	0.8	0.8
M. abscessus subsp. *abscessus* M9	T28	Resistant	0.8	6
M. abscessus subsp. *abscessus* M199	T28	Resistant	0.8	6
M. abscessus subsp. *abscessus* M337	T28	Resistant	0.8	6
M. abscessus subsp. *abscessus* M404	C28	Sensitive	0.2	0.6
M. abscessus subsp. *abscessus* M422	T28	Resistant	0.4	2
M. abscessus subsp. *bolletii* M232	T28	Resistant	0.4	3
M. abscessus subsp. *bolletii* M506	C28	Sensitive	0.1	0.6
M. abscessus subsp. *massiliense* M111	Deletion	Sensitive	0.4	0.4
M. abscessus subsp. *abscessus* K21	C28	Sensitive	0.4	0.4

a MICs are defined as drug concentrations causing 90% growth inhibition compared to untreated control and are means from two independent experiments.

b Described in references 21, 23, and 24.

c *erm*(41), ribosome methylase. T28 sequevars confer inducible clarithromycin (CLR) resistance. C28 and “deletion” sequevars are CLR sensitive (25). CLR was purchased from Sigma-Aldrich and included as a positive control.

**FIG 2:**
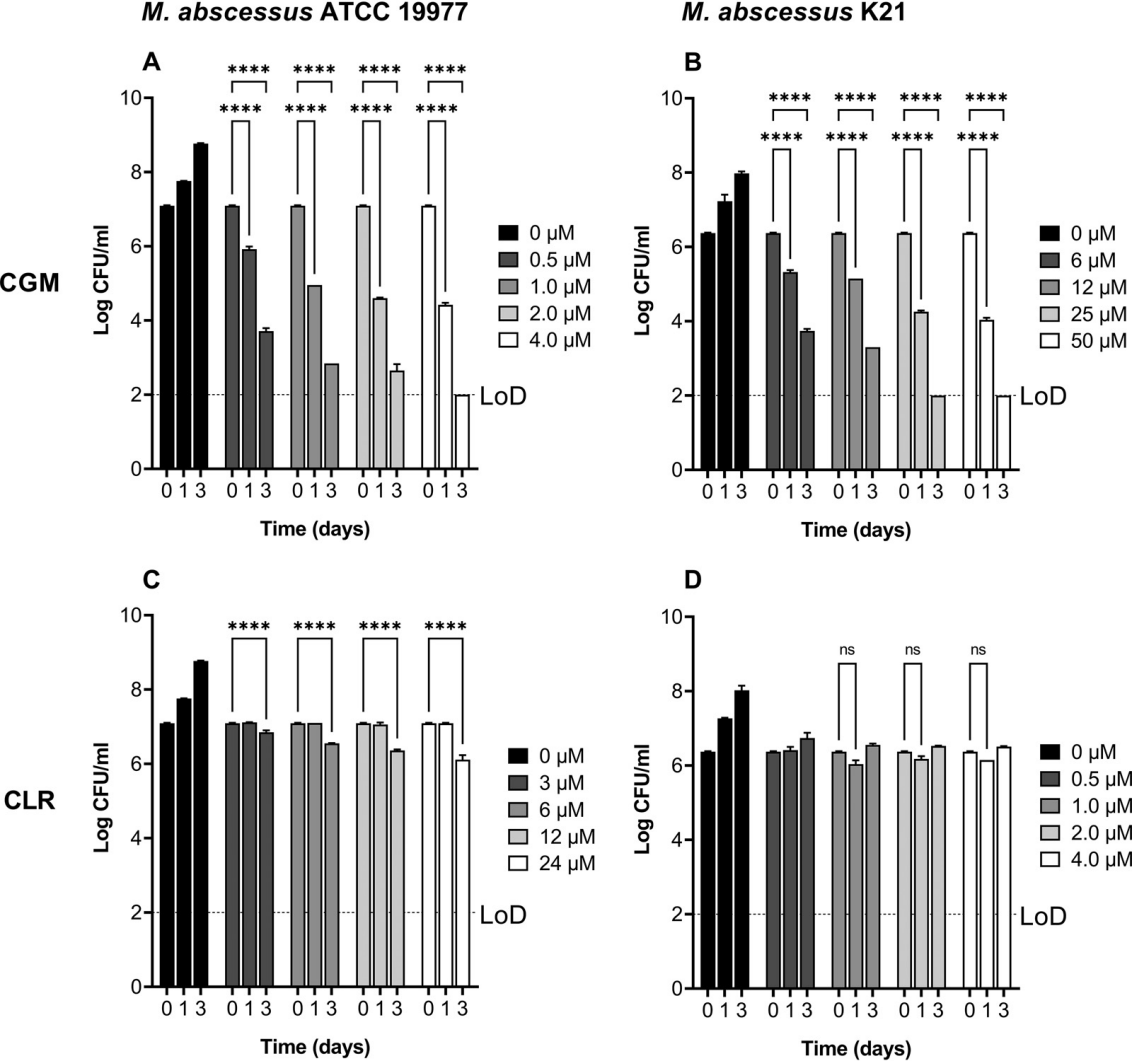
Bactericidal activity of cyclohexyl-griselimycin (CGM) against M. abscessus. (A and B) Exponential-phase cultures of M. abscessus ATCC 19977 (A) and M. abscessus K21 (B) were treated with CGM at the indicated increasing concentrations, and CFU were enumerated at time zero and after 1 and 3 days by plating appropriate sample dilutions on Middlebrook 7H10 agar (BD Difco) (21). CGM MIC against M. abscessus ATCC 19977, 0.5 μM; CGM MIC against M. abscessus K21, 0.4 μM (Table 1). Note the higher CGM concentrations required to reduce the CFU of M. abscessus K21 cultures compared to M. abscessus ATCC 19977. (C and D) Results of the same experiment carried out with the mainly bacteriostatic clarithromycin (CLR) as a control. Note that M. abscessus ATCC 19977 harbors a functional *erm*(41) conferring inducible macrolide resistance (reflected by a higher MIC of 3 μM) (Table 1), whereas M. abscessus K21 harbors a noninducible sequevar of *erm*(41) (reflected by a lower MIC of 0.4 μM) (Table 1). The results were analyzed by two-way analysis of variance (ANOVA) multicomparison (****, *P < *0.0001; ns, nonsignificant [*P > *0.05]). Experiments were carried out three times independently. Mean values and standard deviations are shown. LoD, limit of detection.

To determine whether CGM retained bactericidal activity as observed against M. tuberculosis (12), dose-response time-kill experiments were carried out with the type strain M. abscessus ATCC 19977 (21). Treatment with CGM at the MIC (0.5 μM) resulted in 10-fold and >1,000-fold reductions in CFU after 1 and 3 days, respectively, indicating pronounced time-dependent bactericidal activity (Fig. 2A). Time kill experiments were also carried out for M. abscessus K21, the strain we employ in our mouse infection studies (see below). Interestingly, the bactericidal activity of CGM against M. abscessus K21 was lower than that against M. abscessus ATCC 19977. Despite showing similar MICs against both strains (∼0.5 μM) (Table 1), higher concentrations of CGM were required to achieve comparable reduction of CFU in M. abscessus K21 cultures (Fig. 2B). The reason for the apparent strain-dependent bactericidal activity of CGM remains to be determined. Figure 2C and D show the results of the time-kill experiments for the mostly bacteriostatic clarithromycin as control. Consistent with previous results (22), treatment with the macrolide did not result in significant reduction of CFU.

**FIG 3:**
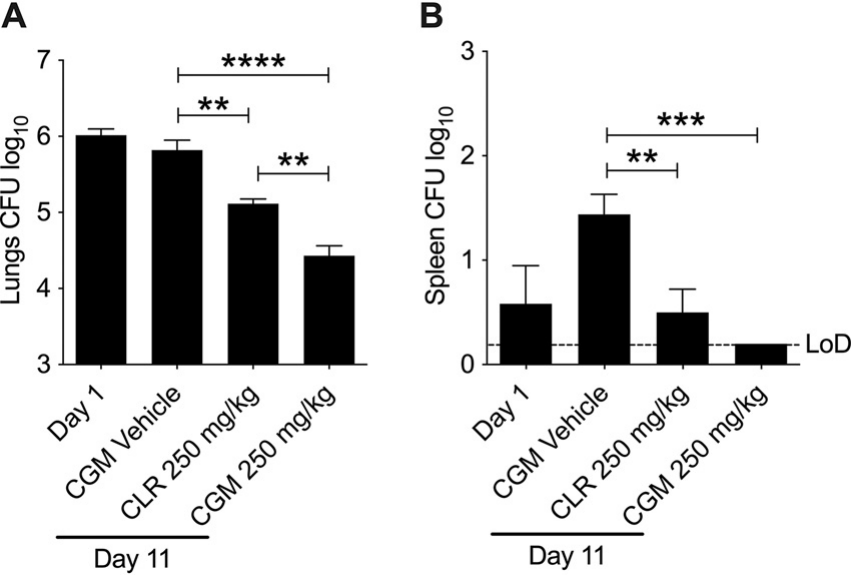
*In vivo* activity of cyclohexyl-griselimycin (CGM) against M. abscessus. NOD SCID mice were infected intranasally with 10^6^ CFU of M. abscessus K21. At 1 day postinfection, 6 mice were euthanized to determine the bacterial load of organs prior to starting chemotherapy. Groups of 6 mice were treated by daily oral gavage for 10 consecutive days with CGM, the control drug clarithromycin (CLR), or vehicle only and were euthanized 24 h after the last dose. The bacterial burden in lungs (A) and spleen (B) was determined by plating organ homogenates on agar. Data were analyzed using one-way analysis of variance (ANOVA) multicomparison and Tukey’s posttest (**, *P < *0.01; ***, *P < *0.001; ****, *P < *0.0001). One representative data set of two independent experiments is shown. LoD, limit of detection. The LoD for the results depicted in panel A was 10 CFU.

To assess the *in vivo* efficacy of CGM, we infected 8-week-old female NOD.CB17-Prkdc^scid^/NCrCrl (NOD SCID) mice (Charles River Laboratories) by intranasal delivery of 10^6^ CFU M. abscessus K21 as described previously (23). In this immunodeficient mouse model, the K21 strain produces a sustained infection resulting in a largely constant bacterial lung burden over time, thus allowing the effects of drugs to be evaluated (23). Drugs or the vehicle control was administered once daily for 10 consecutive days by oral gavage, starting 1 day postinfection. CGM was formulated in Cremophor RH 40–Capryol 90–Miglyol 812 N (10/20/70 [wt/wt/wt]) and administered at 250 mg/kg of body weight. Clarithromycin, formulated in 0.4% methylcellulose–sterile water, was used as a positive control at the human-equivalent dose of 250 mg/kg. All mice were euthanized 24 h after the last dose, and bacterial load in the lungs and spleen was determined by plating serial dilutions of the organ homogenates onto Middlebrook agar. All experiments involving live animals were approved by the Institutional Animal Care and Use Committee of the Center for Discovery and Innovation, Hackensack Meridian Health. As expected, treatment with the vehicle did not affect the bacterial lung burden (Fig. 3A). Compared to the vehicle control, treatment with 250 mg/kg CGM reduced lung CFU 10-fold and thus more than the positive-control clarithromycin at 250 mg/kg (Fig. 3A). CFU reduction in the spleen followed a similar pattern (Fig. 3B). Thus, CGM is efficacious in a mouse model of M. abscessus infection.

In conclusion, we show that the cyclohexyl analog of griselimycin, CGM, is broadly active against the M. abscessus complex *in vitro*. The advanced anti-TB lead compound displayed bactericidal activity *in vitro* and reduced the bacterial lung burden in a mouse model of M. abscessus infection. This work adds a new advanced lead compound to the preclinical M. abscessus drug discovery pipeline and suggests that the new anti-TB drug candidate could be explored for the treatment of M. abscessus lung disease. The demonstration that yet another TB active displays anti-M. abscessus activity supports the paradigm of exploiting chemical matter generated for TB drug discovery to accelerate *de novo* drug discovery for M. abscessus.
